# Implementation of a Single Quadrupole Mass Spectrometer for Fingerprint Analysis: *Venenum bufonis* as a Case Study

**DOI:** 10.3390/molecules23113020

**Published:** 2018-11-19

**Authors:** Wenlong Wei, Xia Wang, Jinjun Hou, Changliang Yao, Zijin Feng, Jianqing Zhang, Sumei Han, Yanping Deng, Yong Huang, Wanying Wu, Dean Guo

**Affiliations:** 1 School of Pharmacy, China Pharmaceutical University, Nanjing, Jiangsu 210009, China; 13521032532@163.com; 2 National Engineering Laboratory for TCM Standardization Technology, Shanghai Research Center for Modernization of Traditional Chinese Medicine, Shanghai Institute of Materia Medica, Chinese Academy of Science, Shanghai 201203 China; xiawang7788@163.com (X.W.); jinjun_hou@simm.ac.cn (J.H.); cpuyao@126.com (C.Y.); cpufzj@126.com (Z.F.); jianqingzhang0928@hotmail.com (J.Z.); ylhwssy@126.com (S.H.); shimbiro@sina.com (Y.D.); hydess@163.com (Y.H.)

**Keywords:** mass spectrometry fingerprint, *Venenum bufonis*, quality control, chemometrics analysis

## Abstract

The mass spectrometry (MS) has been widely used for profiling chemical components of traditional Chinese medicine (TCM). However, there are few studies reporting quality control of TCM based on mass spectrometry fingerprint (MSF) due to its complicated operation and high cost. The aim of this study was to extend the application of MSF for quality evaluation of TCM. In this study, an MSF based on single quadrupole mass spectrometry method was established, and was successfully used for the quality control of *Venenum bufonis* (VB), a famous TCM which was used clinically for cancer treatment in China. The results showed that the superiority of MSF for more chemical information exposure and the finding of more potential chemical markers (eight versus four) compared with the traditional photo-diode array (a kind of ultra violet detector, PDA). Besides, the performance of MSF was also validated by similarity and principle component analysis (PCA) of MS data acquired on two other mass spectrometry (low-resolution, triple quadrupole, QQQ, and high-resolution, quadruple time-of-flight, Q-TOF), showing high consistency with QQQ and Q-TOF, but robustness with few parameters’ settings. Based on our study, MSF could be widely applied for the quality control of TCM.

## 1. Introduction

Fingerprints occupy an important position in the assessment and quality control of complex analytes, especially for traditional Chinese medicines (TCMs). It has been internationally accepted by the Word Health Organization, US Food and Drug Administration, and China Food and Drug Administration [1]. Combining with multivariate statistical analysis, fingerprints can reveal the quality information of TCMs, including common or characteristic chemical components, as well as their respective ratios, which could ensure product quality consistency [2,3]. Up to now, chromatography fingerprint techniques are mainly conducted on the platform of thin layer chromatography (TLC), high/ultra-high performance liquid chromatography (HPLC/UHPLC), hydrophilic interaction chromatography (HILIC), and gas chromatography (GC) [4]. As is widely known, each platform has its own respective pros and cons.

Flexibility, easy to use, low cost, and high speed are the major advantages of TLC. Parameters, such as sample application, plate development, documentation, and derivatization can be optimized easily. However, the frequent use of TLC is often hampered due to its low reproducibility, resolution, sensitivity, efficiency, and accuracy [5,6]. On the contrary, HPLC is more widely used for its high resolution, sensibility, and accuracy [7]. Additionally, its strong separation ability often performs in an automatable manner, which makes the operation more convenient. Nevertheless, extended analytical time, inability to withstand high pressure, and large use of solvent are considered its imperfections. To cover these shortages of HPLC, UHPLC was introduced subsequently. In the price of high cost and high requirement of the sample preparation, UHPLC has showed faster separation and less consumption of solvent with increasing resolution and sensibility when combing with sub-2 μm packing particles [8]. Whereas, reversed-phase liquid chromatography (RPLC), the most popular mode in HPLC/UHPLC, shows its prominent performance only in the separation of nonpolar or medium-polar compounds. When it comes to the separation of polar compounds, co-elution and weak retention are always observed in RPLC. Fortunately, HILIC provides an alternative solution for normal-phase chromatography (NPLC). Using water and organic as the mobile phase, HILIC exhibits professional and environmentally friendly performance in the separation of polar compounds [9]. As for GC, it focuses on separation and identification of volatile components, for example, essential oils [10]. High-resolution and sensitive data is available when GC is coupled with kinds of detectors (FID, MS). A brief table containing advantages and drawbacks of the analytical tools mentioned above are summarized in the supporting information (Table S1). So far, HPLC coupled with ultra violet (HPLC-UV) is used most frequently in fingerprint analysis. However, due to the limits of UV detector, HPLC-UV based fingerprint can only expose compounds with UV absorbing, neglecting lots of non-UV absorbing components. Although some fused fingerprint strategies have been employed recently to capture the chemical information of TCMs as much as possible [11,12], the analysis process is still performed in a complicated mode.

Rapid development of mass spectrometry marked a new stage in the quality control of TCMs. Due to its higher selectivity and sensitivity, LC/MS has been applied widely for qualitative and quantitative analysis of TCMs [13,14]. On one hand, high resolution mass spectrometry (HRMS) cooperated with database retrieval and screening strategies accelerates the global profiling of natural products [8], unveiling their chemical components. On the other hand, the employment of low mass spectrometry in the authentication of TCMs with durability, repeatability, and low analysis cost makes it an available choice for quality control of TCMs [15].

*Venenum Bufonis* (VB, Chinese name “Chan Su”), the dried secretions of the auricular and skin glands of *Bufo gargarizans* Cantor or *Bufo melanostrictus* Schneider, is a well-known TCM that has been broadly used clinically as a cardiotonic, diuretic, anodyne, and antineoplastic drug [16,17,18]. Bufadienolides and alkaloids are the primary constituents. It has been reported that bufadienolides possess strong cardiotoxicity, which is similar to that of digitalis, exhibiting bradycardia, atrioventricular conduction blockage, ventricular tachycardia, and even leading to sudden death [19,20,21,22], while the alkaloids constituents are related with hallucinogenic actions. Thus, quality control of VB is extremely important. According to the Chinese Pharmacopoeia (2015 version), the quality of VB is assessed by HPLC-UV determination of the total contents of cinobufagin and resibufogenin (not less than 6%). Nevertheless, note that surrogates or counterfeits still occur frequently in the market with the presentence of adulteration, thus enhanced quality control methods of VB are in great need.

**Figure 1:**
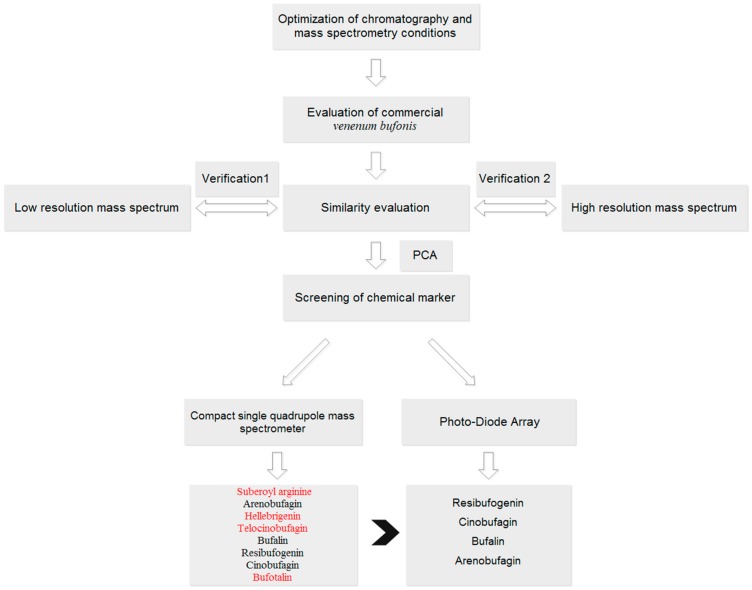
A general workflow for implementation of a compact single quadrupole mass spectrometer for fingerprint analysis: *Venenum bufonis* as a case study.

The aim of this study was to develop a fingerprint based on single quadrupole mass spectrometry for quality control of TCMs. Twenty-eight batches of VB were used as a case study (Figure 1). A traditional PDA (UV detector) based fingerprint was used to evaluate the performance of MSF by the comparison of the number of common peaks, chemometrics analysis, and screening of potential chemical markers. Furthermore, data acquired on two other mass spectrometry (low-resolution, triple quadrupole, QQQ, and high-resolution, quadruple time-of-flight, Q-TOF) were also analyzed to validate the performance of MSF. Finally, the results demonstrated the superiority of MSF compared with UPLC-PDA based fingerprints for more component information exposure, and the consistency and robustness of single quadrupole mass spectrometry (QDA) compared with QQQ and Q-TOF for similar performance with less parameter settings and low analysis cost. Currently, the available literature with respect to fingerprint analysis is mostly focused on UV detectors. Although the concept of MSF has already been mentioned before, it was used for the description of chemical profiling, which was conducted in a single sample. To the best of our knowledge, few studies have conducted analysis of MSF based on batches of samples, nor have they done MSF based similarity analysis and chemometrics analysis. Furthermore, this study did systematic comparison between MSF and UV based fingerprints to evaluate these two detectors’ performance, which has not been reported before. Verifications of two other mass spectrometry (low- and high-resolution) were also performed. Combining with the results of comparison and verifications, we recommend the use of a single quadrupole mass spectrometry for quality control study of TCM in the future, which is conducted in an easy-to-use and economic way.

## 2. Results and Discussion

### 2.1. Optimization of HPLC Chromatographic Conditions

The simultaneous separation of alkaloids and bufadienolides was challenging due to their chemical diversity and heterogeneity. The choice of packing material of chromatographic column was crucial to resolve this puzzle. Five C18 mechanism columns (Figure S1), including Kinetex XB-C18 (2.1 × 100 mm, 1.7 μm), CSH C18 (2.1 × 100 mm, 1.7 μm), HSS T_3_ (2.1 × 100 mm, 1.8 μm), XBridge C18 (2.1 × 100 mm, 3.5 μm), and BEH C18 (2.1 × 100 mm, 1.7 μm), and five HILIC mechanism columns (Figure S2), including BEH HILIC (2.1 × 100 mm, 1.7 μm), BEH amide (2.1 × 100 mm, 1.7 μm), Luna HILIC (2.0 × 150 mm, 3 μm), Kinetex HILIC (2.1 × 100 mm, 1.7 μm), and Xbridge Amide (2.1 × 150 mm, 3.5 μm), were screened and evaluated firstly. Due to the lack of separation efficiency and peak capacity, the HILIC columns were ignored. Compared with the C18 columns, the HSS T3 column was eventually chosen for its better separation and resolution of the majority of peaks. The gradient elution (acetonitrile and 0.1% formic acid-water) showed a great influence on the retention times of analytes, separation efficiency, and peak capacity. So, the gradient systems of the mobile phase were gradually tried and optimized for the separation of coelutions or isomeric peaks (Figure S3). The addition of formic acid was advantageous for optimizing the ionization response and peak tailing. Thus, the performance of different concentrations (0.05%, 0.1%, and 0.5%) of formic acid was evaluated (Figure S4), and the 0.1% concentration was used. In addition, to obtain satisfactory chromatographic conditions, the flow rates (0.3, 0.4, and 0.5 mL/min) and column temperatures (30, 35, 40, and 45 °C) were compared in detail (Figures S5 and S6). The optimized flow rate was 0.5 mL/min and column temperature was 30 °C. Chromatograms of the present sample are shown in Figure S7.

### 2.2. Quality Assurance/Quality Control

Quality assurance (QA) and quality control (QC) are vital parts of all analytical procedures. These practices make the data obtained from experiments stronger and more reliable. Thus, it is necessary to fulfill these procedures.

All the vials used for LC-MS analysis were baked for 1 h at 600 °C, and were washed with deionized water and methanol to remove possible background contamination. A blank sample was prepared using the same chemical reagents with samples. Before each sequential analysis, an equal and zero volume of blank sample were injected, respectively, to check whether there was some presence of interfering substances in the chemical reagents or mobile phase. Nevertheless, neither of them presented detectable interfering substances.

Six reference standards (Arenobufagin, bufotalin, cinobufotalin, Bufalin, cinobufagin, and resibufogenin) were selected as certified reference materials to verify the reliability of instruments and the applicability of the analytical procedures. Repeatability was assessed by analyzing six replicates of the six reference standards. The results showed that the relative standard deviation (RSD) of the retention time and peak area obtained on QDA were in the range of 0.06–0.2% and 2.66–3.91%, respectively. Intermediate precision was carried out by analyzing the reference standards on three consecutive days. The result showed that the RSD of the retention time and peak area obtained on QDA were in the range of 0.09–0.18% and 3.50–5.69%, respectively. Reproducibility (inter-laboratory) was conducted by six replicates of each reference standard in two laboratories. The RSD of the retention and peak area were 0.07–0.1% and 2.14–5.57%, respectively.

Furthermore, S14 was selected as a representative sample to monitor the system stability. It was inserted in every five sample injections in the data acquisition procedure. The RSD of the retention time and peak area of five peaks (retention time of 6.74, 7.74, 11.48, 12.04, and 15.16) were in the range of 0.06–0.12% and 1–3%, respectively. The results indicated that the instrument was stable and the data quality was reliable. Method detection limits (MDLs) and method quantitation limits (MQLs) were calculated to achieve the signal to noise ratio (S/N) of 3 and 10. As a result, the MDLs of the six references were in the range of 1–2 µg/mL. The MQLs of six reference standards were all approximately 10 µg/mL.

### 2.3. Comparison of Fingerprints and Similarity Analysis among Different Detectors

**Figure 2:**
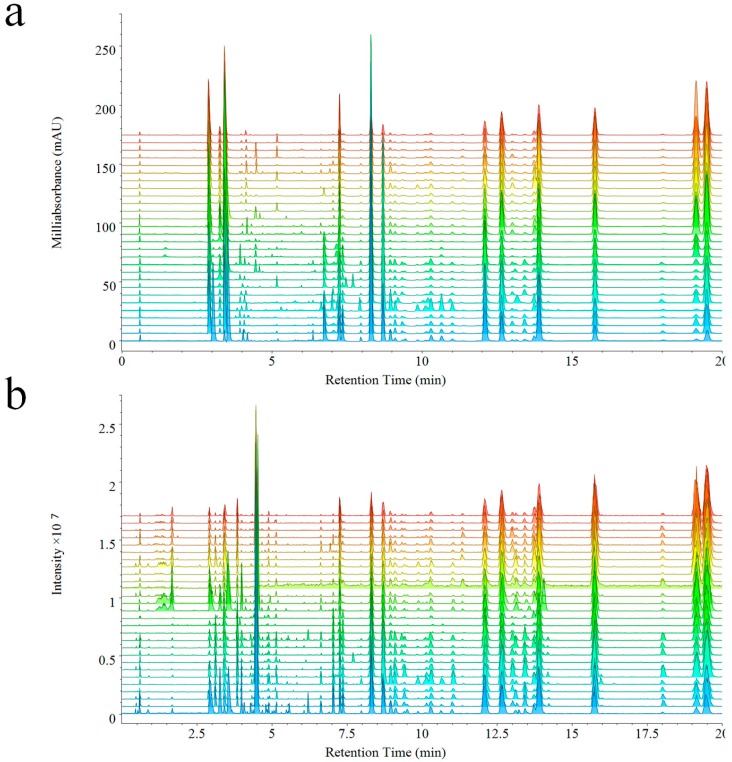
Fingerprints of 28 batches of VB based on PDA (**a**) and QDA (**b**).

The analysis of 28 batches of VB performed by UPLC-PDA-QDA, PDA, and QDA detector based fingerprints were generated at the same time (Figure 2a,b). As expected, components detected by QDA were distinctly more than PDA. In detail, the chemical information between 1–2 min was missed in PDA, but was detected by QDA due to its ability of detecting components without ultraviolet absorption. Furthermore, a higher intensity was also observed in QDA during different elution times (such as, 4–7 min, 13–13.5 min, 18–18.5 min) thanks to its higher sensitivity. The MSF gave much convenience to the analysis of fingerprints, which could make extraction of special peaks on the fingerprint. Though complicated compounds existed in the extract of VB, compounds of different types could be extracted and displayed directly from the fingerprint of QDA. The molecular weights of most alkaloids were among 150–380 Da, and the molecular weights of bufadienolides were among 380–800 Da (including free type and dicarboxylic acid-conjugated type: 380–650 Da, arginine-conjugated type: 650–800 Da). The extracted ion chromatograms are shown in Figure S8.

**Figure 3:**
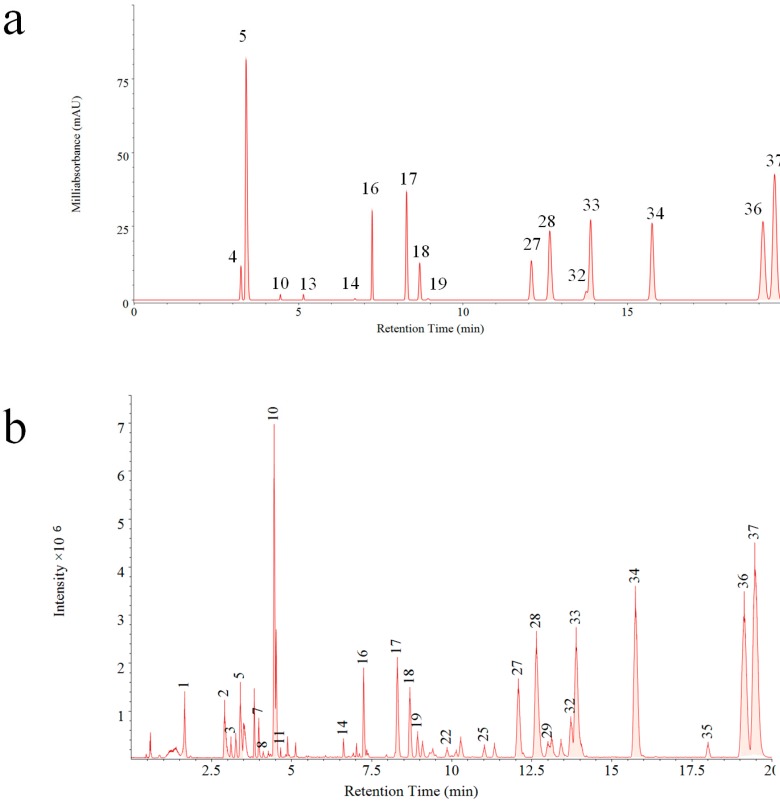
Common peaks based on PDA based fingerprint (**a**) and MSF (**b**).

A reference fingerprint is often generated after analysis of dozens of samples with information of common fingerprint peaks, which display the main components in a holistic aspect. In this study, both PDA and QDA based reference fingerprints were generated (Figure 3). Significant differences can be visually compared between two reference fingerprints. Thirty-seven common peaks (Figure 3b) were formed by fingerprints obtained on QDA, which was about 1.3 times more than that of PDA (16 common peaks, Figure 3a). This result indicated the superiority of MSF for more chemical component information exposure as well as evaluation of VB in a comprehensive mode.

Compared with the PDA detector, the additional mass information of the fingerprint of VB could be obtained easily by QDA. Characterization of 37 common peaks detected by UPLC-QDA from VB are listed in Table 1. To further comprehensively confirm these peaks, the parent ions and MS/MS fragmentations of the detected peaks were acquired on UPLC-Q-TOF/MS. Molecular formulas were formed based on accurate mass measurement with a mass error of less than 5 ppm. As a result, 12 compounds were identified or tentatively characterized.

**Figure 4:**
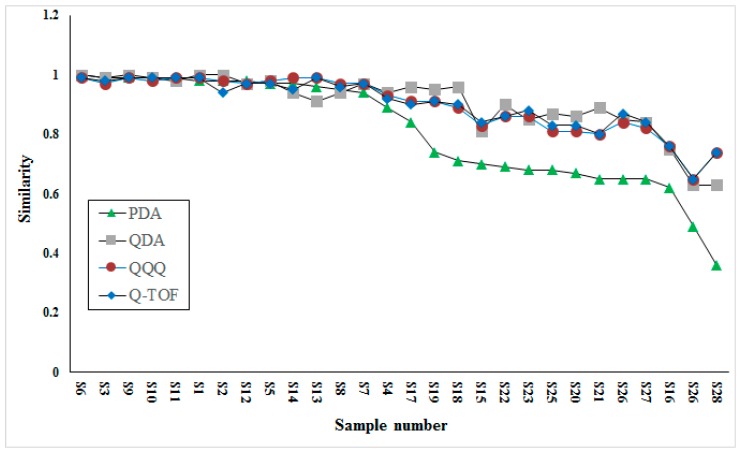
Similarities analysis of data acquired on four different detectors.

The comparison of similarity analysis between QDA and PDA detectors based fingerprints was conducted to investigate the different performances. Besides, similarity analysis based on two other MS detectors (low-resolution, QQQ; high-resolution, Q-TOF) were also conducted to validate the performance of QDA. The comparison result is shown in Figure 4. Interestingly, the results acquired on three MS detectors were quite similar for their higher similarity in most samples, while results acquired on PDA were apparently different from others for its lower similarity in half of samples. Taking detectability into consideration, we speculated that this result may be caused by many minor compounds, for example, components eluted during 1–2 min, which were important for the similarity contribution of VB, but were neglected by the PDA detector due to its detector restriction (low sensitivity and requirement for ultraviolet absorption). Through the comparison of similarity among three MS detectors, the result acquired from QDA possessed high consistency with two others.

### 2.4. Fingerprint Based Chemometrics Analysis

**Figure 5:**
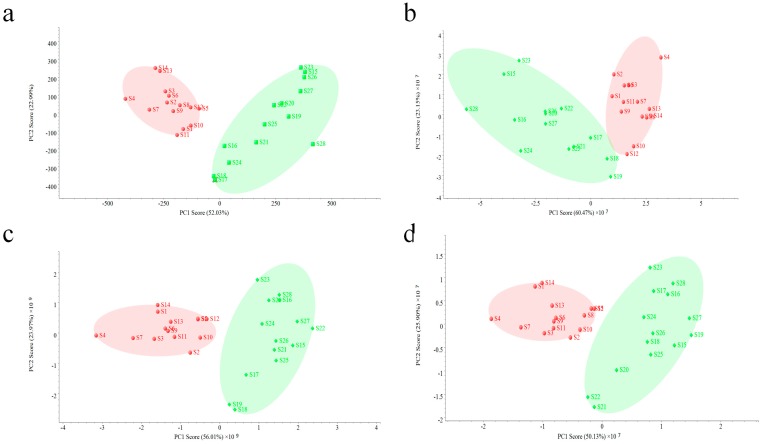
PCA analysis of data acquired on four different detectors (**a**–**d**) represents results based on PDA, QDA, QQQ, and Q-TOF, respectively).

An unsupervised PCA model was used for the further evaluation of two fingerprints. Data acquired on two other MS detectors were also used to validate the performance of QDA. As shown in Figure 5, all PCA analysis results based on four detectors were quite similar, making all samples clearly divided into two groups, but small difference can still be observed. The interval of the PCA result based on PDA (Figure 5a) was bigger than others (Figure 5b–d), which indicates that PDA did much better in the classification of all VB samples. However, in this study, reasons causing this result should be concerned. Since the PDA data used in the PCA model was related with 16 common peaks in all VB samples, which were in a narrow aspect, a small change of variables might cause a big difference. On the contrary, PCA analysis of MS data was performed in a more comprehensive way, and due to its employment of more related component information (37 common peaks), the difference among batches of samples could be observed in a holistic aspect, making the evaluation result more reliable.

**Figure 6:**
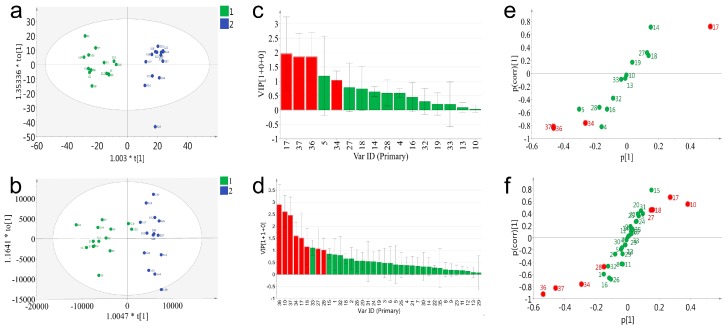
OPLS-DA analysis of 28 batches of VB samples based on PDA and QDA detectors. (**a**) The score scatter plot of PDA, (**b**) the score scatter plot of QDA, (**c**) the VIP pot of PDA, (**d**) the VIP pot of QDA, (**e**) the S-plot of PDA, and (**f**) the VIP pot of QDA.

To deeply exploit and compare the data acquired on PDA and QDA, a supervised statistical model, orthogonal projection of latent structures-discriminant analysis (OPLS-DA), was applied for the distinction of the samples and screening for potential chemical markers. All batches of VB were classified into two groups in advance according to the previous result of PCA. It was reported that the accumulated values of Q2 (cum), >0.3 possessed statistically significant, >0.5 represented good, and >0.9 indicated that the model was perfect [23]. As shown in Figure 6, both PDA and QDA based OPLS-DA model (Figure 6a) showed good fitness (R2 = 0.85, Q2 = 0.82 for PDA and R2 = 0.84, Q2 = 0.8 for QDA). The PDA and QDA results of the summary of fit showed good fitness and predictive performances, and the model revealed a small cross-validation between R2 (cum) and Q2 (cum) values. The permutation strongly supported the validity of the OPLS-DA model (Figure S9a,b), since the Q2 regression line possessed a negative intercept and all permuted R2-values (green color in the left) were lower than the original point of the R2-value (green color in the right).

To explore the potential markers that contributed to classification of 28 batches of samples both PDA and QDA based variable importance projection (VIP) values were calculated and used to pick out the discriminatory variable (Figure 6c,d). The variables with a VIP ≥ 1 were considered to be more relevant for sample classification. Subsequently, four (peak 17, 37, 36, 34) and nine (peak 36, 10, 37, 34, 17, 18, 33, 27, 28) potential markers were identified with satisfactory deviations (peak 5 in Figure 6c was eliminated for its high deviations) in the PDA and QDA based models, respectively. In order to further verify this result, an S-plot was conjunctively analyzed to examine the OPLS-DA predictive component loading to facilitate model interpretation as well as find the potential markers that were responsible for the discrimination of the classified groups. As shown in Figure 6e,f, all the variables were distributed into either the first or the third quadrant, indicating the model was well established. The red points meeting the requirements of both VIP ≥ 1 and shifting from others were considered the potential markers. Particularly, although the VIP value of peak 33 in Figure 6d was large than 1, the location of peak 33 was in the center of the coordinate axis in the S-plot. Thus, peak 33 was not considered as a potential marker. Eventually, based on the results of the VIP value and S-plot, four potential markers (peak 17, 34, 36, 37) in the PDA based model and eight (peak 10, 17, 18, 27, 28, 34, 36 and 37) in QDA were selected. Notably, QDA performed better in the number of found potential markers. Potential markers (peak 17, 34, 36, 37) found by PDA could also be found by QDA, which indicated the partial consistency of the results between these two detectors. Besides, four additional markers (peak 10, 18, 27, 28) found by QDA further represented the superiority of QDA. The extraction ion chromatograms of eight characteristic peaks are described in Figure S10, and the chemical structures of eight characteristic constituents are shown in Figure S11. Amongst these potential markers, 36 and 37 (resibufogenin and cinobufagin) were the significant compounds whose content are under strict control in Chinese Pharmacopoeia (2015 edition; total contents of cinobufagin and resibufogenin: Not lower than 6%). Furthermore, one of the four additional markers, peak 10, represented suberoyl arginine, which is an alkaloid in VB. As previous study has reported that these types of compounds were related with hallucinogenic action, it is thus necessary to include them in quality control criterion.

## 3. Materials and Methods

### 3.1. Chemicals and Reagents

Two bufadienolides compounds, resibufogenin and cinobufagin, were isolated from VB in our laboratory, and their structures were identified by detailed high-resolution MS and NMR analysis. These two compounds showed purity >98% as determined by HPLC-UV. HPLC-grade acetonitrile, methanol (Merck, Darmstadt, Gemany), and formic acid (Sigma-Aldrich, St Louis, MO, USA), and deionized water (18.2 MΩ at 25 °C) prepared by a Millipore Alpha-Q water purification system (Millipore, Bedford, MA, USA), were tested or used in the mobile phase for chromatographic separation of VB extracts. Information regarding the origin of VB materials (28 batches) analyzed in this study is available in the Supporting Information, Table S2.

### 3.2. Sample Preparation

An easy-to-implement ultrasonic extraction method was employed. Briefly, 25 mg accurately weighted fine powder of VB was ultrasonically extracted in 5 mL methanol on a water bath (1130 W, 37 kHz) at room temperature for 30 min. Then, 1 mL extracting solution was transferred to a 1.5-mL centrifuge tube, followed by centrifugation at 14,000 rpm for 10 min. The obtained supernatant was prepared as the sample solution for QDA analysis, and was diluted 100-fold for QQQ and Q-TOF analysis. All the sample solutions obtained were stored at 4 °C prior to analysis.

### 3.3. UPLC Conditions

Chromatographic separation was performed on a Waters ACQUITY I-Class UPLC^®^ system (Waters Corporation, Milford, MA, USA) equipped with a binary solvent manager, a sample manager, a column manager, and a PDA detector. An ACQUITY UPLC^®^ HSS T3 column (Waters Corporation, Milford, MA, USA) (1.8 μm, 2.1 × 100 mm) equipped with an on-line filter was used and eluted by a binary mobile phase composed of acetonitrile (B) and 0.1% formic acid (*v*/*v*; A) following a gradient elution program: 0–2 min: 5% B; 2–4 min: 5–17% B; 4–6 min: 17–28% B; 6–9 min: 28–28% B; 9–14 min: 28–35% B; 14–20 min: 35–39% B; 20–21 min: 39–95% B; 21–25 min: 95–95% B. The column temperature was set at 30 °C. The flow rate was 0.5 mL/min, and 1 µL of the test solution was injected.

### 3.4. Mass Spectrometry Conditions

Low resolution MS data were recorded on a single quadruple MS detector (Waters Corporation, Milford, MA, USA) QDA at a low cone voltage of 15 V with a sample frequency of 8 Hz operating in the positive ion mode. The mass analyzer scanned over a mass range of 150–800 Da. The mass spectrometry conditions of Q-TOF-MS and QQQ-MS are provided in the supporting information.

### 3.5. Data Pretreatment and Multivariate Statistical Analysis

QDA Centrodied MS data acquisition and processing were conducted by Empover^®^ 3 (Waters, Manchester, UK), while QQQ and Q-TOF were conducted by MassLynx V4.1 software (Waters, Manchester, UK).

Raw data of multi-batches of VB samples were uploaded into ChemPattern software (2017, Chemmind Technologies Co., Ltd., Beijing, China) to perform chromatographic peak alignment, data normalization, and similarity evaluation using the default setting. The obtained data matrix involving t_R_, *m*/*z*, and normalized peak area was imported into SIMCAP-P 14.1 software (Umetrics, Umea, Sweden) for chemometric analysis. The components showing a VIP value ≥1 were considered as potential markers.

## 4. Conclusions

In this study, two kinds of fingerprints (PDA based and QDA based, respectively) were compared in terms of the number of common peaks, chemometrics analysis, and screening of potential chemical markers, using 28 batches of VB as a case study. Sixteen common peaks were detected by PDA based fingerprint versus 37 common peaks by MSF. This result showed the superiority of QDA for more components’ information exposure, the finding of more potential markers, thanks to its outstanding performance on the detection of components with UV absorbing and non-UV absorbing. Data obtained by two other MS detectors (low-resolution, QQQ and high-resolution, Q-TOF) were also analyzed; similar results were observed in similarity and PCA analysis. With the increasing requirement for more sensitive and accurate analysis, high-resolution mass spectrometry, e.g., Q-TOF, was frequently used in qualitative analysis, and low-resolution, but with enhanced scan methods, e.g., QQQ, was widely used in quantitative analysis. However, both were conducted at the expense of complicated parameter settings (see Supporting Information) and high analysis cost. Conversely, QDA was performed in an easier mode with only few parameter settings (see Section 3.4) and low analysis cost. Thus, this study confidently demonstrated the consistent, but robust, performance of QDA. Finally, according to the S-plot and VIP value analysis, four potential chemical markers (VIP ≥ 1) were found by PDA based fingerprint, while eight (four same markers with PDA and four additional markers) were found by MSF. One of the four additional chemical markers, suberoyl arginine, belongs to alkaloids, which was in the absence of quality control of VB, but related with biological activity. It is not surprising that the number of potential chemical markers found by MSF was more than that found by PDA due to its superior detecting ability. However, it is notable to realize the overlapping results between MSF and PDA based fingerprints. In other words, potential chemical markers found by PDA could also be found by QDA, indicating the partial consistency of behavior between these two detectors. Moreover, the additional markers added confidence to the employment of QDA. Chemical ingredients play a vital role in disease treatment, thus it is quite necessary to be aware of them as much as possible, especially in the quality control process. Taken together, and considering the chemical diversity of TCMs or natural products, we recommend the broad application of single quadrupole mass spectrometry based MSF for future quality control, which is conducted in a more historic aspect with the advantage of ease of use, less parameter settings, and low analysis cost.

## Figures and Tables

**Table 1:** Characterization of chemical compounds by UPLC-QDA and UPLC-Q-TOF/MS from VB.

NO.	Tr (min)	QDa (*m*/*z*)	Q-TOF (*m*/*z*)	Product Ion	Q-TOF (ppm)	Formula [M + H]^+^	Identification
**Alkaloids**							
**1**	1.65	275	275.1357	257.1235	0.7	C_10_H_19_N_4_O_5_	Unknown
**3**	3.07	303	303.1671	250.1181	1	C_12_H_23_N_4_O_5_	Unknown
**5**	3.37	219	219.1497	160.08	−0.5	C_13_H_19_N_2_O	Bufotenidine
**6**	3.5	347	347.1931	294.4285; 219.1490	−0.2	C_14_H_27_N_4_O_6_	Unknown
**7**	3.81	317	317.1825	158.09; 175.12; 264.14	2.2	C_13_H_25_N_4_O_5_	Pimeloyl arginine
**8**	3.94	203	203.1144	173.0709; 118.0649	3.8	C_7_H_15_N_4_O_3_	Unknown
**9**	4.3	326	326.1716	124.0865	−2.5	C_15_H_24_N_3_O_5_	Unknown
**10**	4.42	331	331.1981	278.15; 158.09; 250.16	1.2	C_14_H_27_N_4_O_5_	Suberoyl arginine
**11**	4.65	345	345.2138	159.0663	3.5	C_15_H_29_N_4_O_5_	Unknown
**12**	4.87	359	359.2294	241.0692; 116.0694	−2.5	C_16_H_31_N_4_O_5_	Unknown
**13**	5.13	345	345.2138	302.0553; 112.0870	0.8	C_15_H_29_N_4_O_5_	Unknown
**14**	6.61	575	575.3121	352.1914; 241.0693	0.5	C_34_H_43_N_2_O_6_	Unknown
**26**	11.33	643	643.3694	337.1077; 275.1344	−1.3	C_34_H_51_N_4_O_8_	Unknown
**30**	13.1	715	715.4281	421.1974; 331.1978	−0.1	C_38_H_59_N_4_O_9_	Unknown
**35**	17.97	699	699.4349	536.6514; 150.9849	0.3	C_39_H_55_N_8_O_4_	Unknown
**Bufadienolides**							
**15**	7.02	417	417.2208	399.2163	0.3	C_24_H_33_O_6_	Iso-arenobufagin
**16**	7.33	417	417.2271	399.2173	−0.6	C_24_H_33_O_6_	Iso-arenobufagin
**17**	8.27	417	417.2280	399.22; 363.19; 335.20	0.3	C_24_H_33_O_6_	Arenobufagin
**18**	8.68	417	417.2274	363.19; 335.20	−0.3	C_24_H_33_O_6_	Hellebrigenin
**19**	8.91	417	417.2268	363.19; 335.20	−0.9	C_24_H_33_O_6_	Iso-hellebrigenin/arenobufagin
**20**	9.07	403	403.2483	385.24; 367.23; 349.22; 271.21	−0.1	C_24_H_35_O_5_	Iso-telocinobufagin
**21**	9.33	459	459.2386	363.1957; 301.1436	0.3	C_26_H_35_O_7_	Hydroxycinobufagin
**22**	9.82	415	415.2215	337.1052; 317.1823	−0.6	C_24_H_31_O_6_	Unknown
**23**	10.12	403	403.2484	331.1976; 301.1424	0.0	C_24_H_35_O_5_	Unknown
**24**	10.27	473	473.2164	367.1902; 349.1817	-1.1	C_26_H_33_O_8_	Iso-19-oxocinobufotalin
**25**	10.99	401	401.2317	347.1964; 301.1416	-1.1	C_24_H_33_O_5_	Iso-desacetylcinobufagin
**27**	12.08	403	403.2484	385.24; 367.23; 349.22; 271.21	0.1	C_24_H_35_O_5_	Telocinobufagin
**28**	12.65	445	445.2597	385.24; 367.23; 349.22; 331.21	0.7	C_26_H_37_O_6_	Bufotalin
**29**	13.00	399	399.2168	331.1969	-0.3	C_24_H_31_O_5_	Unknown
**31**	13.40	457	457.2226	397.2018; 333.1857	0.0	C_26_H_33_O_7_	Iso-19-oxocinobufagin
**32**	13.72	401	401.2322	365.2052; 337.1026	-0.6	C_24_H_33_O_5_	Iso-marinobufagin
**33**	13.89	459	459.2383	381.21; 363.20; 345.18	0.5	C_26_H_35_O_7_	Cinobufatalin
**34**	15.75	387	387.2535	351.23; 333.22; 255.21	-0.2	C_24_H_35_O_4_	Bufalin
**36**	19.14	385	385.2368	367.23; 321.22;271.20; 253.20	-1.1	C_24_H_33_O_4_	Resibufogenin
**37**	19.47	443	443.2434	365.21; 347.20; 329.19	0.2	C_26_H_35_O_6_	Cinobufagin

## Supplementary Materials

The following are available online. Figure S1: Optimization of columns of C18 mechanism, Figure S2: Optimization of columns of HILIC mechanism, Figure S3: Optimization of gradient elution, Figure S4: Optimization of concentration of formic acid, Figures S5,6: Optimization of gradient elution, Figure S7: Chromatogram of a representative sample by UPLC-PDA (**a**)-QDa (**b**), Figure S8: Extraction of different mass ranges (**a**): 150–380 Da, (**b**): 380-650 Da, (**c**): 650–800 Da, Figure S9: Permutation test of OPLS-DA of PDA (**a**) and QDA (**b**), Figure S10: Extraction of eight characteristic peaks, Figure S11: The chemical structures of seven characteristic constituents, Table S1: Comparison of different analytical tools, Table S2: The information of the 28 batches of VB samples.

## Abbreviations

MS	mass spectrometry
TCM	traditional Chinese medicine
MSF	mass spectrometry fingerprint
VB	Venenum bufonis
QQQ	triple quadrupole mass spectrometry
Q-TOF	quadruple time-of-flight mass spectrometry
