# Supplementary material for: Implementation of a Single Quadrupole Mass Spectrometer for Fingerprint Analysis: Venenum bufonis as a Case Study
Source: Molecules. 2018 Nov 19;23(11):3020. doi: 10.3390/molecules23113020 (PMC6278474; doi:10.3390/molecules23113020)
Supplement: Supplementary file 1 [file molecules-23-03020-s001.pdf]

Implementation of a single quadrupole mass spectrometer for fingerprint analysis: *Venenum bufonis* as a case study

Wenlong Wei [1,2,†], Xia Wang [2,†], Jinjun Hou [2,†], Changliang Yao [2], Zijin Feng [2], Jianqing Zhang [2], Sumei Han [2], Yanping Deng [2], Yong Huang [2], Wanying Wu [2,*], Dean Guo [2,*]

1 School of Pharmacy, China Pharmaceutical University, Nanjing, Jiangsu 210009, China; 13521032532@163.com

2 National Engineering Laboratory for TCM Standardization Technology, Shanghai Research Center for Modernization of Traditional Chinese Medicine, Shanghai Institute of Materia Medica, Chinese Academy of Science, Shanghai 201203 China; jinjun_hou@simm.ac.cn; (J.H.); xiawang7788@163.com (X.W.); cpuyao@126.com (C.Y.); cpufzj@126.com (Z.F.); jianqingzhang0928@hotmail.com (J.Z.); ylhwssy@126.com (S.H.); shimbiro@sina.com (Y.D.); hydess@163.com (Y.H.)

* Correspondence: wanyingwu@simm.ac.cn (W.W.); daguo@simm.ac.cn (D.G.); Tel.: +86-21-20231000 (ext.2221) (W.W. & D.G.); Fax: +86-21-50272789 (W.W. & D.G.)

† These two authors contributed equally to this work.

Received: 8 October 2018; Accepted: 13 November 2018; Published: date

Supporting Information

Mass Spectrometry conditions of Q-TOF-MS and QQQ-MS

The MSFs obatanind on Q-TOF-MS and QQQ-MS were used to compare the consistency of MSF obtained on ACQUITY QDa detector. The same chromatographic condition as that used in UPLC-PDA-QDa was applied. Positive mode in mass range of *m*/*z* 150–800 was adopted at both the two MS instruments. For Waters Xevo G2-S Q-TOF mass spectrometer (Waters Corporation, Milford, MA, USA), capillary voltages of 2 kV , cone voltage of 40 V, cone gas flow of 30 L/h, source temperature of 140 ℃, and desolvation gas flow of 700 L/h at 500 ℃ were utilized. A solution of leucine-enkephalin (1 μg/mL) was used as lock mass for data calibration. For Xevo QQQ micro mass spectrometer (Waters Corporation, Milford, MA, USA), capillary voltages of 3 kV and cone voltage of 30 V was set. Source temperature was 148 ℃, and desolvation temperature was 597 ℃. Cone gas flow was 50 L/h, and desolvation gas flow was 947 L/h. Data acquisition and processing were performed using MassLynx V4.1 software (Waters Corporation, Milford, MA, USA).

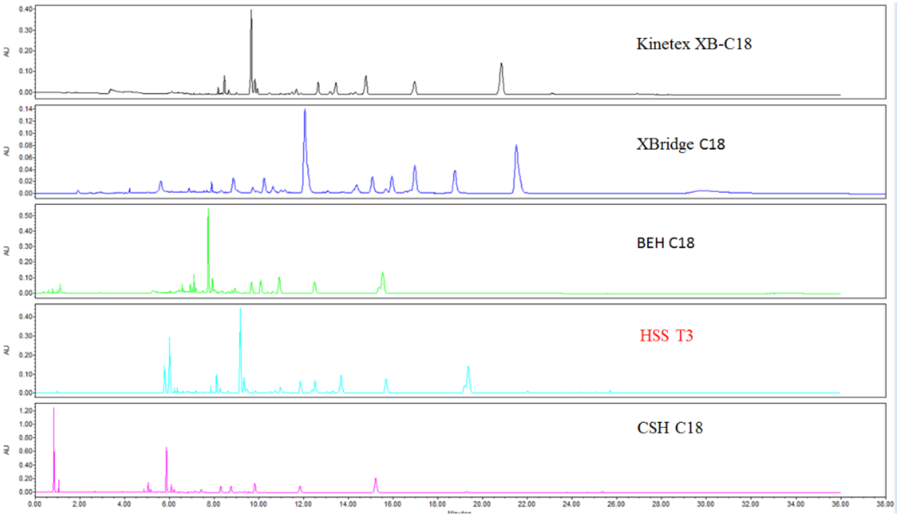

Figure S1. Optimization of columns of C18 mechanism.

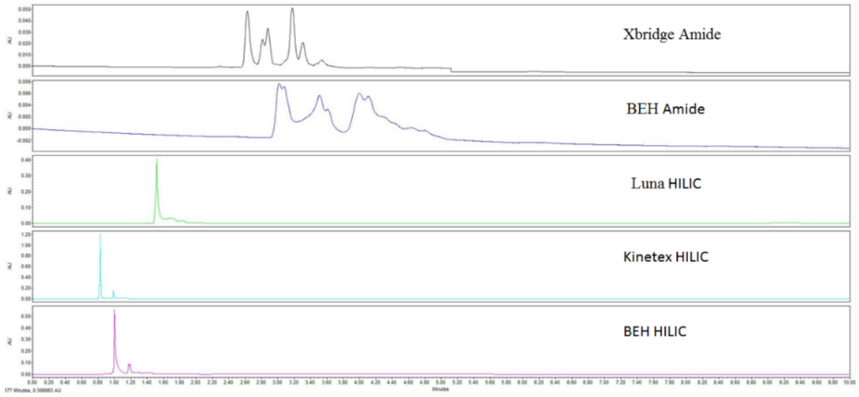

Figure S2. Optimization of columns of HILIC mechanism.

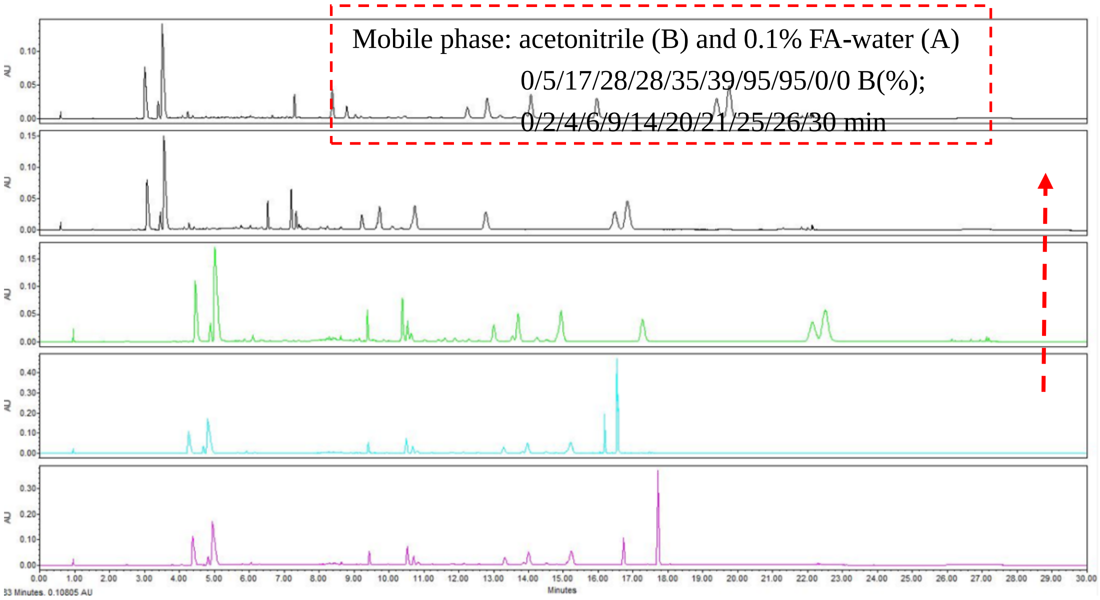

Figure S3. Optimization of gradient elution.

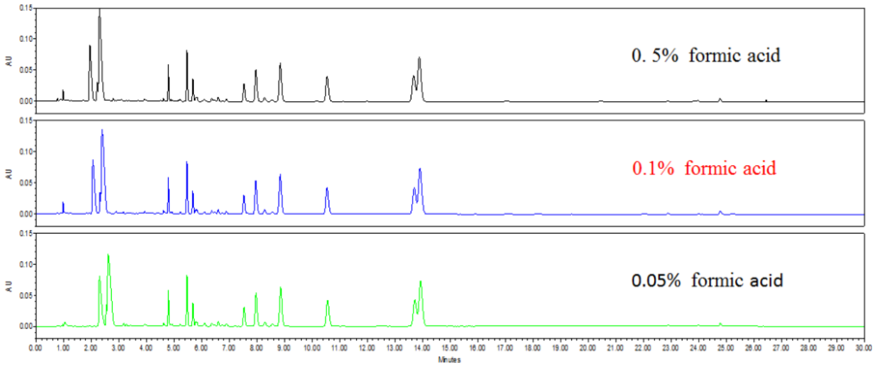

Figure S4. Optimization of concentration of formic acid.

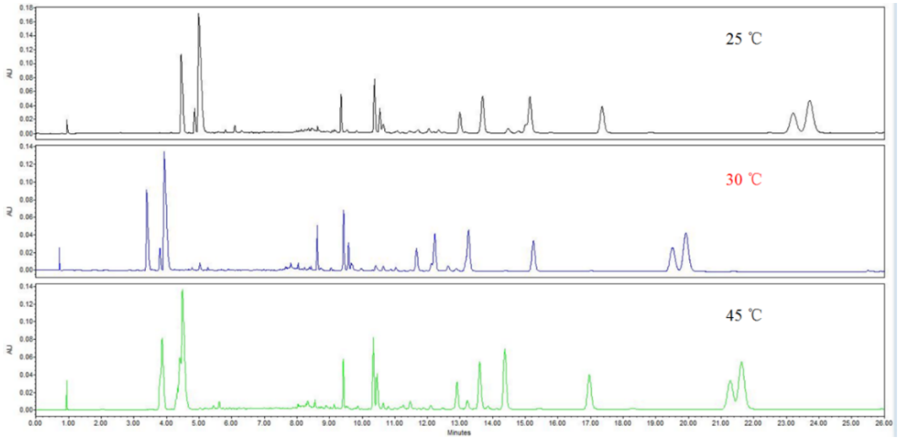

Figure S5. Optimization of gradient elution.

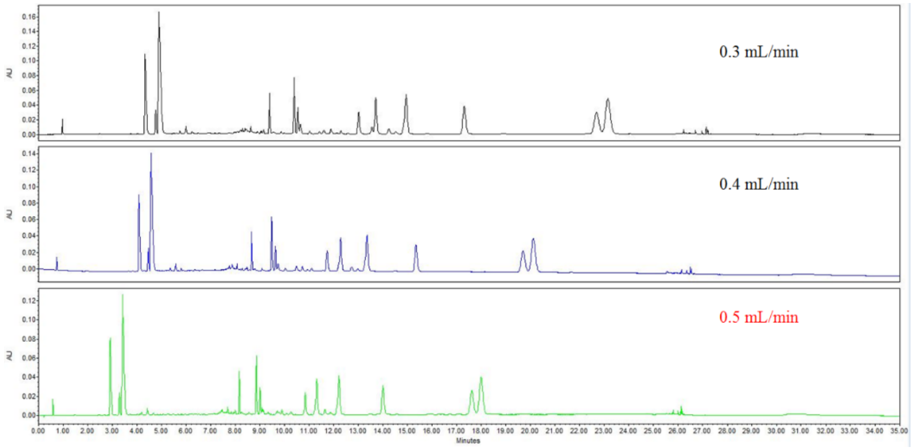

Figure S6. Optimization of gradient elution.

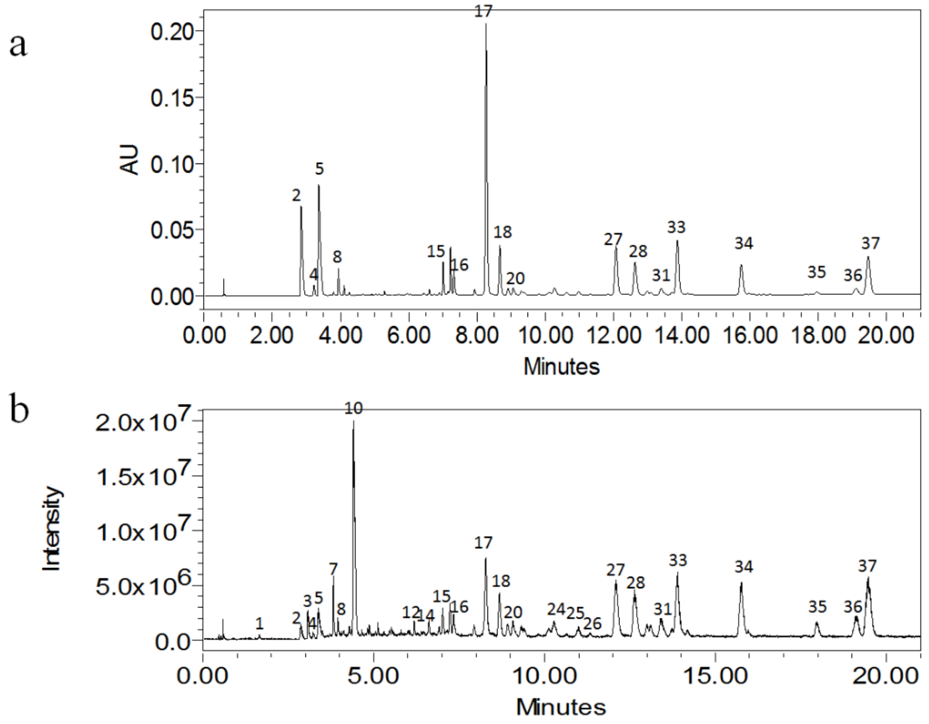

Figure S7. Chromatogram of a representative sample by UPLC-PDA (**a**)-QDa (**b**).

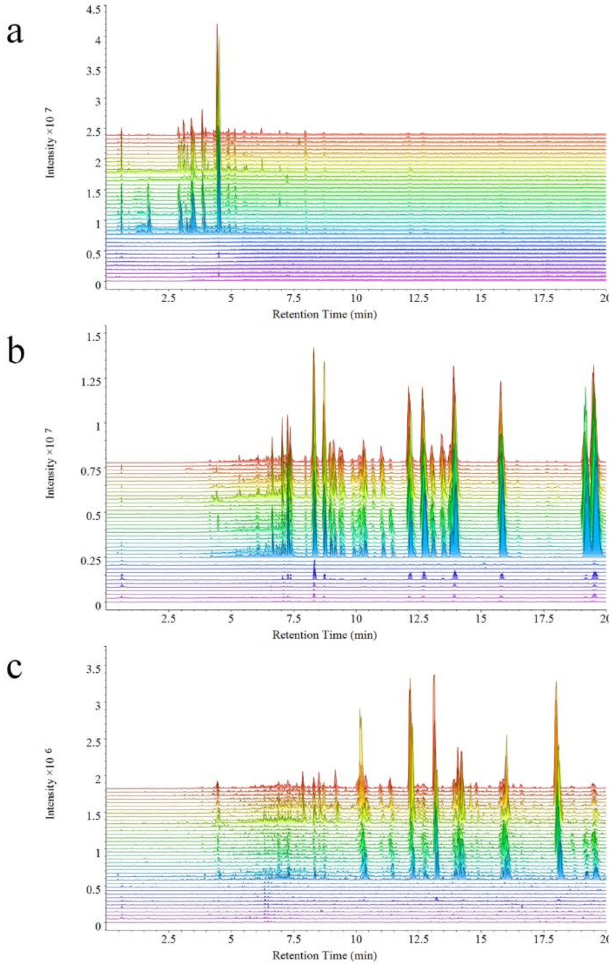

Figure S8. Extraction of different mass ranges (a: 150-380 Da, b: 380-650 Da, c: 650-800 Da)

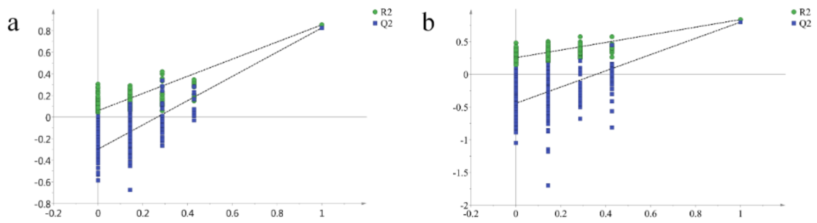

Figure S9. permutation test of OPLS-DA of PDA (**a**) and QDA (**b**).

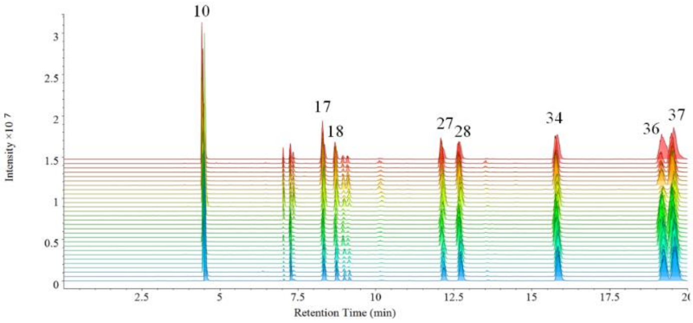

Figure S10. Extraction of eight characteristic peaks.

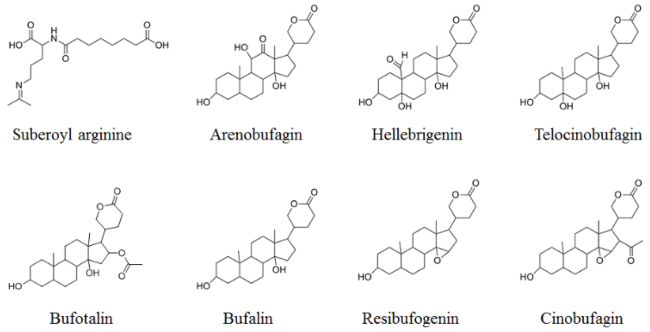

Figure S11. The chemical structures of seven characteristic constituents.

Table S1. Comparison of different analytical tools.

	Advantages	Drawbacks
**Separation**		
TLC	High flexibility	Low reproducibility and resolution
	Easy to use	Low sensitivity
	Low cost and high speed	Low efficiency in separation
		Low accuracy in quantification
HPLC	Automatable operating	Extended analytical time
	High resolution, sensitivity and accuracy	Requirement for large volume of solvent
UHPLC	High resolution separation	Sample preparation have high requirement
	Reduced analytical time	
	Reduced solvent consumption	
HILIC	Suitable for polar compounds	Narrow range of application
	Environmentally friendly mobile phase	
GC	High sensitivity	Limited to volatile compounds
	High resolution	Thermal instability
**Detection**		
MS	High sensitivity	Not easy operation
	Powerful component characterization	Low precision
UV	Simple operation	Low component characterization
	Wide linear range	Low sensitivity
	High precision	

Table S2. The information of the 40 batches of VB samples.

No.	Provider	Production Region	Descriptio n	Collection Time
**S15**	Yongfa Chanchu breeding bases, Changyi district, Jilin	Jilin	Slice	04/2014
**S1**	Bozhou medicine market	Unknown	Slice	04/2014
**S2**	Bozhou medicine market	Unknown	Slice	04/2014
**S3**	Bozhou medicine market	Shandong	Slice	05/2014
**S4**	Bozhou medicine market	Shandong	Slice	06/2014
**S5**	Web of Chinese herbal medicins	Unknown	Slice	04/2014
**S6**	XingWang Chanchu breeding bases	Unknown	Slice	04/2014
**S7**	Anguo of Hebei	Bozhou	Nubble	09/2014
**S8**	Anguo of Hebei	Bozhou	Nubble	09/2014
**S9**	Anguo of Hebei	Bozhou	Nubble	09/2014
**S10**	Yinfa, Linyi	Sichuan	Nubble	09/2014
**S11**	Yinfa, Linyi	Jilin	Nubble	09/2014
**S12**	Yinfa, Linyi	Shandong	Nubble	09/2014
**S13**	Songjialiang, Linyi	Shandong	Nubble	09/2014
**S14**	Anguo of Hebei	Bozhou	Nubble	09/2014
**S15**	Bozhou medicine market	Shandong	Slice	04/2014
**S16**	Guangming pharmacy, Anguo of Hebei	Jilin	Slice	04/2014
**S17**	Bozhou medicine market	Henan	Slice	04/2014
**S18**	Shaanxi medicine market	Sichuan	Nubble	08/2014
**S19**	Songjialiang, Linyi	Shaanxi	Nubble	09/2014
**S20**	Guangming pharmacy, Anguo of Hebei	Jilin	Nubble	04/2014
**S21**	Guangming pharmacy, Anguo of Hebei	Jilin	Slice	04/2014
**S22**	Bozhou medicine market	Shandong	Slice	04/2014
**S23**	Bozhou medicine market	Unknown	Slice	04/2014
**S24**	Shaanxi medicine market	South America	Nubble	08/2014
**S25**	Yulin medicine market	Bozhou	Slice	08/2014
**S26**	Yinfa, Linyi	Anhui	Nubble	09/2014
**S27**	Yinfa, Linyi	Shandong	Nubble	09/2014
**S28**	Songjialiang, Linyi	Sichuan	Nubble	09/2014
